# Small DNA Pieces in *C. elegans* Are Intermediates of DNA Fragmentation during Apoptosis

**DOI:** 10.1371/journal.pone.0011217

**Published:** 2010-06-18

**Authors:** P. Joseph Aruscavage, Sabine Hellwig, Brenda L. Bass

**Affiliations:** 1 Department of Biochemistry, University of Utah, Salt Lake City, Utah, United States of America; 2 Department of Microbiology and Molecular Genetics, University of Pittsburgh School of Medicine, Pittsburgh, Pennsylvania, United States of America; University of Texas MD Anderson Cancer Center, United States of America

## Abstract

While studying small noncoding RNA in *C. elegans*, we discovered that protocols used for isolation of RNA are contaminated with small DNA pieces. After electrophoresis on a denaturing gel, the DNA fragments appear as a ladder of bands, ∼10 nucleotides apart, mimicking the pattern of nuclease digestion of DNA wrapped around a nucleosome. Here we show that the small DNA pieces are products of the DNA fragmentation that occurs during apoptosis, and correspondingly, are absent in mutant strains incapable of apoptosis. In contrast, the small DNA pieces are present in strains defective for the engulfment process of apoptosis, suggesting they are produced in the dying cell prior to engulfment. While the small DNA pieces are also present in a number of strains with mutations in predicted nucleases, they are undetectable in strains containing mutations in *nuc-1*, which encodes a DNase II endonuclease. We find that the small DNA pieces can be labeled with terminal deoxynucleotidyltransferase only after phosphatase treatment, as expected if they are products of DNase II cleavage, which generates a 3′ phosphate. Our studies reveal a previously unknown intermediate in the process of apoptotic DNA fragmentation and thus bring us closer to defining this important pathway.

## Introduction

Programmed cell death (PCD), or apoptosis, is triggered by external stimuli such as viral infection, but is also a normal part of homeostasis and an animal's development into an adult. Studies in *C. elegans* were key in elucidating three genetically distinguishable events in apoptosis during which a cell is instructed to undergo PCD (“specification”), initiates the apoptotic program (“killing”) and finally, degrades its components and is engulfed by neighboring cells (“execution”; reviewed in [1], [2]).

DNA degradation during the execution phase is initiated by nucleases in the apoptotic, dying cell, and completed by lysosomal nucleases of the neighboring cells that engulf the dying cell (reviewed in [3]). Two intermediates of apoptotic DNA degradation have been observed, with high molecular weight fragments (HMW, 50–300 kbp) temporally preceding those of low-molecular weight (LMW, ≥180 bp; [4]). The existence of intermediates indicates DNA degradation does not occur simply from release of lysosomal enzymes into the dying cell [5], since the latter would release both proteases and nucleases and result in a smear of DNA cleavage fragments as observed during necrosis. Rather, the HMW and LMW fragments indicate that nucleases act on DNA that is still protected by chromatin, with HMW fragments presumably reflecting nuclease accessibility of higher-order chromatin organization (e.g., loops; see [4]) and LMW fragments that of a nucleosomal, beads on a string organization. LMW intermediates can be monitored by non-denaturing electrophoresis, and are evidenced by a ladder of DNA fragments that are multiples of ∼180 bp, indicative of internucleosome cleavage. DNA fragmentation has also been monitored by the appearance of free 3′ hydroxyls, which can be labeled using the terminal deoxynucleotidyl transferase (TdT) dUTP-mediated end labeling (TUNEL) protocol.

Numerous nucleases have been implicated in PCD (reviewed in [3], [6], [7]). The best characterized is caspase-activated nuclease (CAD), also called DNA fragmentation factor (DFF). CAD/DFF is a magnesium-dependent endonuclease that catalyzes double-stranded breaks that generate 5′ phosphates and 3′ hydroxyls, and thus, its products are TUNEL reactive. In vertebrate cells, CAD/DFF likely plays a role in generating both HMW and LMW fragments within the dying cell. However, in the absence of CAD, internucleosomal fragments are still produced, at least in part by endonuclease G (endo G; [8]), which also generates fragments with 3′ hydroxyls amenable to reactivity with TdT [9]. In *C. elegans*, which lacks a CAD homolog, an endo G enzyme (CPS-6) contributes to LMW fragmentation [10].

Smaller intermediates in the DNA fragmentation process have not been observed. However, the final steps of degradation occur after engulfment, via lysosomal enzymes, and if DNA from the dying cell is no longer protected by chromatin, intermediates might not accumulate. DNase II endonucleases are thought to play a role in degradation after engulfment, and as is typical of lysosomal enzymes, exhibit an acidic pH optimum (4.5–5.5; reviewed in [6]). DNase II enzymes catalyze single-stranded nicks to generate fragments with 3′ phosphates, which cannot be labeled with the TUNEL protocol. Somewhat surprisingly, in *C. elegans*, the DNase II enzyme NUC-1 plays a role in DNA degradation within the apoptotic cell [11]. Animals containing mutations in *nuc-1* accumulate TUNEL positive material in cells undergoing apoptosis during embryogenesis, suggesting that *nuc-1* acts downstream to convert TUNEL positive fragments to TUNEL negative fragments. How a lysosomal enzyme might act in the apoptotic cell is unclear, although recent studies raise the possibility that NUC-1 is taken up by the dying cell after secretion by other cells [12].

In preparing small noncoding RNAs using conventional protocols, we discovered that these protocols also enrich for small DNA pieces. Investigations aimed at determining the derivation of these small DNA pieces revealed that they were previously unrecognized intermediates in the apoptotic pathway. Analysis of a series of *C. elegans* mutant strains indicates the intermediates are produced by NUC-1, a DNase II endonuclease.

## Results

### Protocols optimized for extraction of RNA also enrich for small DNA pieces

Analyses of small RNAs such as miRNAs and siRNAs typically start with an organic extraction of the biological sample, often using commercially available reagents such as Trizol (Invitrogen). Such reagents are optimized for retention of RNA in the aqueous layer and proteins and DNA in the interface or organic layer. After alcohol precipitation of the aqueous fraction, the RNA is electrophoresed on a high percentage polyacrylamide gel, followed by northern analysis with a radioactive probe complementary to the sequence of the small RNA of interest.

**Figure 1:**
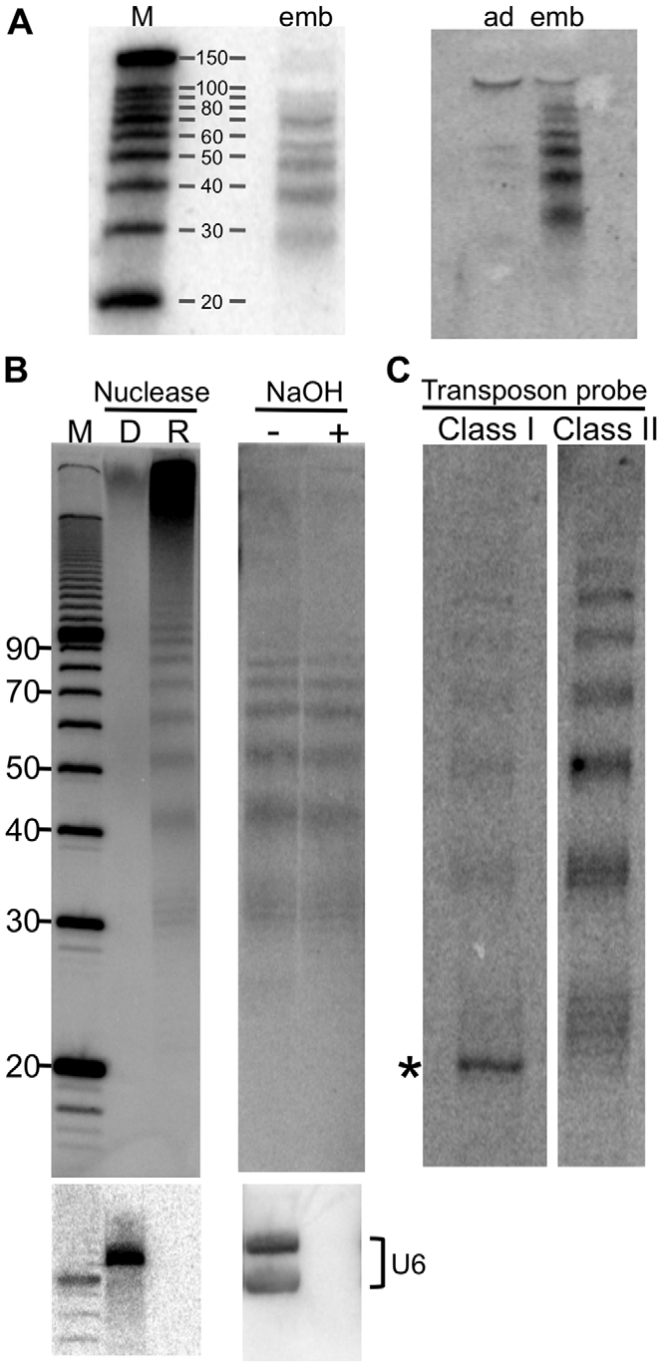
Characterization of small nucleic acid pieces. (**A**) Northern analysis of nucleic acid isolated from wildtype embryos (emb) or adults (ad) using the “total RNA” protocol, and hybridization with probes complementary to the 3′ UTR of *clec-67* (Materials and Methods; Figure S1). M, RNA decade markers (Ambion). (**B**) A representative northern blot is shown (n≥3) preformed as in (A) using embryo samples and subjecting “total RNA” to various treatments prior to electrophoresis and northern analysis: D, DNase treatment; R, RNase treatment, NaOH, alkaline hydrolysis. M, 10 bp DNA ladder (Invitrogen). Blots were reprobed for U6 snRNA to confirm specificity of various treatments (bottom panels). In different experiments, U6 snRNA migrated either as a single or double band. (**C**) A representative northern blot is shown (n≥2) performed as in (A) for embryo “total RNA” using probes for Class I or Class II transposons (see Materials and Methods). Asterisk marks an RNase sensitive band. ImageQuant software was used to calculate the relative radioactivity hybridizing to Class 1 and Class II transposons (1∶4.4); radioactivity in the entire lane, excluding the RNase sensitive band, was evaluated.

In the course of applying such protocols to the identification of small RNAs corresponding to genes regulated by *C. elegans* Dicer [13], we noticed that certain probes gave rise to a ladder of bands by northern analysis, rather than the expected single band of ∼20–25 nucleotides (nts). For example, Figure 1A shows blots using a combined set of 7 radiolabeled oligonucleotide probes, each 50 nts in length, that together encompass 687 nt of the 3′ terminus of the *clec-67* gene (Figure S1). Further characterization showed that the bands were much more abundant in samples prepared from *C. elegans* embryos compared to those of adults (Figure 1A, right panel); in addition, we found that the ladder of bands was identical using probes designed to detect sense or antisense sequence (Materials and Methods; Figure S2).

**Figure 2:**
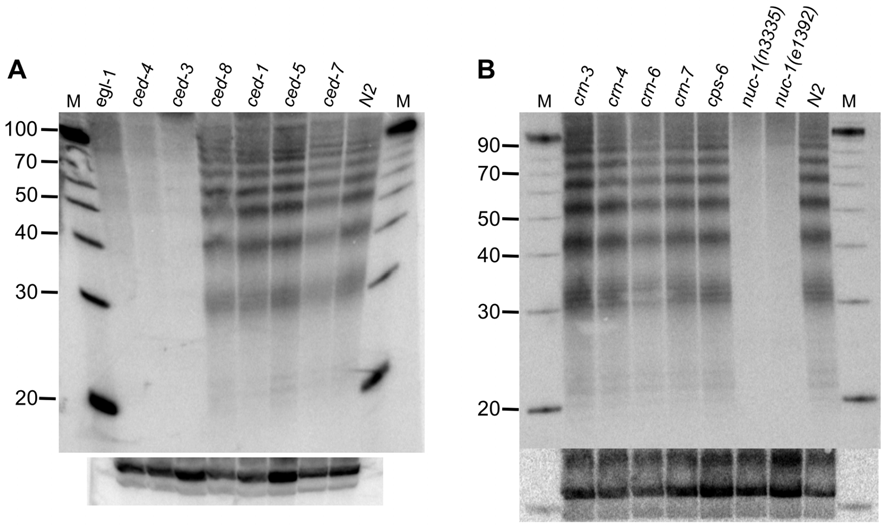
Analysis of small DNA pieces in *C. elegans* apoptosis mutant strains. Northern analyses as in Figure 1 were performed on “total RNA” isolated from *C. elegans* embryos of wildtype (N2) animals or those containing mutations in genes that affect the killing or engulfment phase of apoptosis (A) or those implicated in nucleolytic degradation of DNA during apoptosis (B). For each strain, multiple northern analyses (2–5) were performed on multiple, independent RNA preparations (2–5) and representative data are shown. As much as possible, cultures of various strains were maintained at similar developmental stages, but embryos isolated from gravid adults varied slightly in their precise stage. This may explain the variation in levels of small DNA pieces between strains; further, some differences may be due to mutations in apoptosis genes that alter the kinetics of PCD. The blot shows an absence of small DNA pieces in *ced-3(n717)*, and other alleles (*n1286, n2888)* gave identical results (Figure S4). Blots were reprobed for U6 as a loading control (bottom panels).

To further characterize the species giving rise to the ladder of bands, we electrophoresed the samples on longer gels to provide greater resolution, and also subjected the samples to digestion by DNase, RNase, or alkaline hydrolysis, prior to electrophoresis (Figure 1B). Surprisingly, we found that the ladder of bands was eliminated by DNase treatment, but resistant to RNase and alkaline hydrolysis. This suggested the bands represented small DNA pieces, and consistent with this idea, the increased resolution gave a pattern that resembled that of nuclease digestion of DNA wrapped around a nucleosome core [14], [15]. In the latter, clusters of cleavages occur every helical turn (∼10 nt intervals) as the DNA rises to the surface of the nucleosome where it is most accessible. Similarly, we observed clusters of bands at ∼10 nt intervals. In most cases the smallest species migrated at 29–33 nts, but if more material was analyzed, 20–23 nt species could sometimes be detected (e.g., Figure 2B). Taken together, these data indicated that organic extraction protocols optimized for isolation of RNA also enrich for small DNA pieces.

### Small DNA pieces can be detected with probes to repetitive elements

The probes used for the experiments of Figure 1A consisted of a set of 7 oligonucleotides designed to hybridize to the antisense strand of the 3′ terminus of the *clec-67* gene, and we wondered if all or only a subset of these oligonucleotides were responsible for the hybridization signal. We performed additional northern analyses and found that only two of the 7 oligonucleotides were responsible for the hybridization signal (Figure S2; probes *clec-67*UTR1, *clec-67*UTR2). The hybridizing oligonucleotides both encompassed part of a repetitive element, and we wondered if probes to other repetitive elements would allow visualization of the small DNA pieces.

To this end, we preformed additional analyses using probes designed to hybridize either to Class I retrotransposons or Class II DNA transposons (Figure 1C; Materials and Methods). Blots probed with a mixture of 14 radiolabeled oligonucleotides complementary to 7 distinct retrotransposons, or 8 complementary to 4 distinct DNA transposons, both showed a ladder of hybridizing bands similar to that observed with the *clec-67* probes. All bands were sensitive to DNase treatment (Figure S3), except an ∼28 nt band in the retrotransposon hybridizing material (Figure 1C, asterisk; length estimated from RNA markers), which may represent a small RNA that functions in transposon silencing (e.g., see [16], [17]).

Since the probes were different sequences, the hybridization signal for the two classes of repetitive elements could not be directly compared. However, the strength of the hybridization signal was roughly proportional to copy number. For example, based on their known distribution within the *C. elegans* genome [18], we estimated there were 500 elements that would be detected by our probes to the Class I retrotransposons, and 1959 elements that would be detected by our probes to the Class II DNA transposons. The ratio of 500/1959, or 1 retrotransposon element for every 3.9 DNA transposon elements, agrees well with the 1:4.4 ratio for signal intensity quantified from the northern blot (Figure 1C and legend).

### Small DNA pieces are produced during apoptosis

Several of our observations raised the possibility that the small DNA pieces were products of the DNA fragmentation process that occurs during apoptosis. First, the pieces were most readily observed in embryos (Figure 1A), where apoptosis is a normal part of development, and second, the ability to detect the DNA pieces by hybridization correlated with the copy number of the sequence (Figure 1C). The latter suggested that the pieces derived from a global event that affected the entire genome, but was only detectable for highly abundant sequences.

To determine if the DNA pieces resulted from the DNA fragmentation that occurs during apoptosis, we analyzed mutants known to affect apoptosis in *C. elegans* (reviewed in [1], [2]). Genes essential for the killing phase of apoptosis, whose mutation completely eliminates apoptosis, were devoid of the small DNA pieces (Figure 2A). For example, small DNA pieces were absent in strains containing mutations in *ced-3*, the caspase that promotes apoptosis in *C. elegans*, as well as *ced-4*, required for activation of *ced-3* [19]. Similarly, the DNA pieces were absent in strains containing a mutation in *egl-1*, which encodes a BCL-2 homology region 3 domain-containing protein that functions to relieve inhibition of apoptosis by CED-9 [20].

Having established that visualization of the small DNA pieces required a functional apoptotic pathway, we analyzed additional mutants to determine if engulfment was required, and further, if any of the known apoptotic nucleases were required. Small DNA pieces were readily detectable in the engulfment defective strains, *ced-1*, *ced-5*, and *ced-7* (Figure 2A; reviewed in [21]), as well as a strain containing a mutation in *ced-8*, which slows the kinetics of PCD [22]. Similarly, small DNA pieces were observed in a number of strains containing mutations in genes implicated in DNA fragmentation (Figure 2B), including the endo G homolog, *cps-6*, [10] and those identified in an RNAi screen of candidate apoptotic nucleases (*crn-3*, *crn-4*, *crn-6*, *crn-7*; [23]). In contrast, the small DNA pieces were completely absent in two strains containing mutations in *nuc-1*, a *C. elegans* DNase II homolog [11], [24]), indicating NUC-1 is important for the generation of the small DNA pieces.

### Small DNA pieces do not react with terminal deoxynucleotidyltransferase

NUC-1 is a DNase II homolog, and is thus predicted to produce DNA fragments that have a 3′ phosphate, which cannot be labeled with TdT, i.e., they are TUNEL negative. During embryogenesis, TUNEL positive nuclei appear in strains with mutations in *nuc-1* with similar kinetics as observed in wildtype animals [11]). However, while a wildtype embryo has on average 1–2 TUNEL positive nuclei, *nuc-1* embryos show 40–50 TUNEL positive nuclei, the majority of which remain TUNEL positive at least until hatching [11]. These results suggest that NUC-1 is responsible for converting TUNEL positive fragments into those that are TUNEL negative, and in its absence, TUNEL positive nuclei accumulate. Our observation that the small DNA pieces were absent in *nuc-1* mutant strains suggested that they might correlate with the TUNEL negative fragments, and thus be resistant to labeling with TdT.

**Figure 3:**
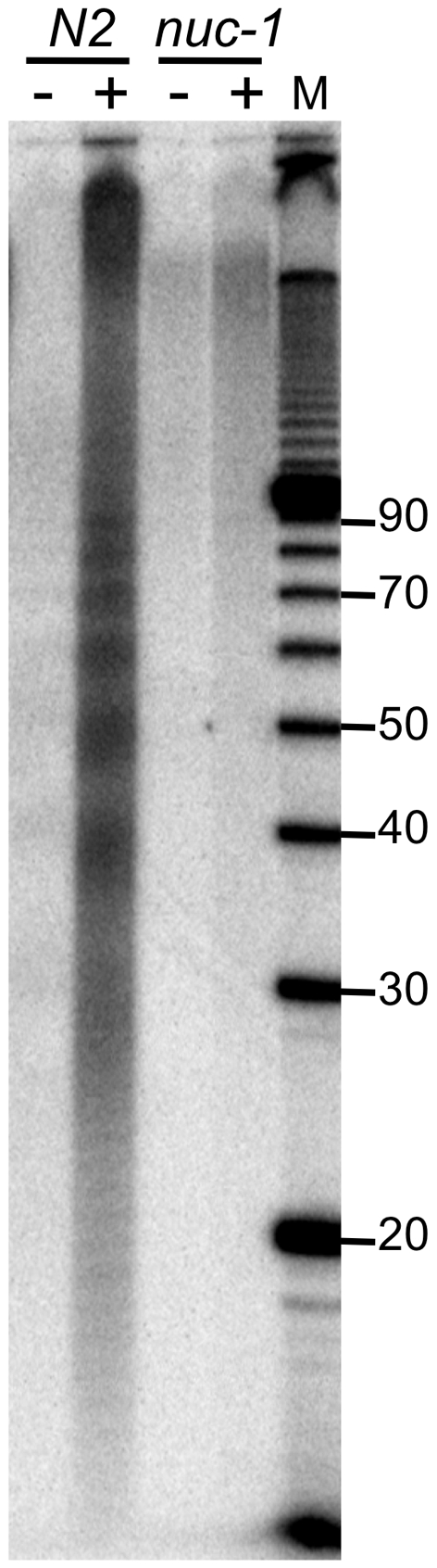
Small DNA pieces correlate with TUNEL negative fragments. Embryos were isolated from gravid adults, of N2 or *nuc-1(e1392)* genotype, and nucleic acid isolated using the “total RNA” protocol. A representative PhosphorImage (n = 7) shows DNA pieces radiolabeled with TdT and α-^32^P-Cordycepin 5′ triphosphate, before (-) or after (+) treatment with CIP. Positions of 10 bp DNA ladder (M) are labeled on the right.

To test this idea we incubated small DNA pieces isolated from wildtype (N2) and *nuc-1* mutant embryos with TdT and α-^32^P-Cordycepin 5′ triphosphate (Figure 3). Embryos were isolated from gravid adults prior to isolation of small DNA pieces, and TdT reactions were performed directly on the nucleic acid (-) or after treating with Calf Intestinal Phosphatase (CIP; +) to remove terminal phosphates. Consistent with the idea that the small DNA pieces observed in wildtype animals have 3′ phosphates, small DNA pieces were not radiolabeled in the absence of CIP treatment. However, after treating with CIP, small DNA pieces were radiolabeled in the wildtype samples, and after electrophoresis the familiar ∼10 nt ladder was observed. Small DNA pieces were not observed in the *nuc-1* mutant sample even after CIP treatment, consistent with the idea that NUC-1 is the enzyme responsible for generating the small DNA pieces. Higher molecular weight labeling was observed in the *nuc-1* sample after CIP treatment, suggesting another enzyme that gives rise to 3′ phosphate fragments also plays a role in DNA degradation during apoptosis in *C. elegans*.

## Discussion

Here we describe a previously unrecognized intermediate in the process of DNA fragmentation during apoptosis. When visualized by electrophoresis on a denaturing gel, the intermediate appears as a ladder of small DNA fragments (≥ 20 nucleotides), roughly spaced at 10 nt intervals. The pattern of DNA fragments is essentially identical to that observed upon nuclease digestion of DNA wrapped around a nucleosome core [14], [15], [25], suggesting the apoptotic nuclease responsible for these fragments targets DNA that is still associated with nucleosomes. Since DNA digestion in the engulfing cell is thought to occur in the presence of proteases that digest chromatin, the small DNA pieces are likely associated with nuclease digestion in the apoptotic cell itself. Importantly, the fragments were absent in *C. elegans* strains containing a mutation in *nuc-1*, which encodes a DNase II enzyme, known to produce fragments with 3′ phosphates. This result, combined with our demonstration that the DNA fragments cannot be labeled with TdT, suggests NUC-1 is the enzyme responsible for producing the small DNA pieces.

### Does NUC-1 act in the apoptotic or the engulfing cell?

Previous analyses of *nuc-1* mutant *C. elegans* show that cell death and cell corpse engulfment occurs in these animals, but the DNA of dead cells is not fully degraded [26], [27]. Further, *C. elegans* strains defective for engulfment (e.g., *ced-1* and *ced-2*) have unengulfed cell corpses containing DNA, indicating that the engulfment process is necessary to complete the DNA degradation process. While these results have been interpreted to mean that engulfment is necessary to activate NUC-1 in the dying cell (e.g., [26]), other studies showed that the ability of NUC-1 to convert TUNEL positive fragments to TUNEL negative fragments is independent of engulfment [11]. Consistent with the latter, we observed that strains defective for engulfment showed normal levels of the small DNA pieces (Figure 2A). Of course, since it is difficult to completely eliminate engulfment in *C. elegans* (reviewed in [21]), it remains possible that some aspect of engulfment is necessary for NUC-1 function.

As previously proposed [6], it is also possible that NUC-1 functions in both the dying and engulfing cell, and this model seems most consistent with existing data. Taking our data into account, in this model NUC-1 would act in the dying cell to cleave DNA that is still protected by nucleosomes into small DNA pieces. Subsequently, after engulfment, proteases would digest the nucleosomes, and NUC-1 would further degrade the small DNA pieces to nucleotides.

### Relationship to previous studies of DNA fragmentation during apoptosis

In *D. melanogaster*, a mutation in the DNase II gene (CG7780) leads to an increase in the DNA fragments that migrate as an ∼180 bp nucleosomal ladder, in both embryos and ovaries [28]. While this accumulation was interpreted to mean the mutant animals had enhanced CAD activity, in light of our results, we would interpret the accumulation as a loss of the downstream DNase II cleavage that converts the ∼180 bp ladder to the smaller ∼10 nt ladder.

Using an RNA interference screen of candidate nucleases, a number of *C. elegans* genes were identified that increase TUNEL positive nuclei during embryogenesis [23]. These genes were named cell-death-related nucleases (*crn*), albeit the nature of the screen did not confirm that the candidate genes are true nucleases. We did not see defects in the production of small DNA pieces in our assays of *crn* mutant strains, which included *crn-6 and crn-7*. Like *nuc-1*, *crn-6* and *crn-7* encode DNase II homologs, and a recent study compared the accumulation of TUNEL positive nuclei in strains containing mutations in these genes [12]. Strains with a mutation in *nuc-1* showed by far the most significant accumulation of TUNEL positive nuclei, but *crn-6* and *crn-7* mutant strains did show a small accumulation compared to wildtype. We did not see residual levels of small DNA pieces in either of two mutant strains of *nuc-1* (Figure 2B). Thus, we conclude that either the contribution of *crn-6* and *crn-7* to the production of small DNA pieces is below our level of detection, or these genes act at a different step of the pathway, possibly in the generation of larger DNA pieces. This idea would be consistent with our observation of higher molecular weight labeling in both wildtype and *nuc-1* samples after CIP treatment (Figure 3).

We also observed small DNA pieces in a strain containing a mutation in the endo G homolog, *cps-6*, also reported to show an increase in TUNEL positive nuclei [10]. CPS-6 yields fragments with 3′ hydroxyls, which are labeled with the TUNEL reaction, and thus it is a bit confusing that loss of this protein leads to an increase in TUNEL positive nuclei. One possibility is that the increase in TUNEL staining observed in these various mutants results from defects in the kinetics and progression of PCD, a phenotype sometimes observed in apoptosis deficient strains (e.g., see [10], [22]).

### Do small DNA pieces contaminate conventional preparations of small RNAs?

Interestingly, none of the protocols in use for preparing small RNAs for deep-sequencing include a DNase step, and thus, the small DNA pieces produced during apoptosis likely contaminate these preparations. However, NUC-1 products have a 3′ phosphate, and thus, in protocols used for cloning the small RNAs that rely on a 3′ hydroxyl, the small DNA pieces would not be included in the cDNA libraries (e.g., [17]). Some protocols include a phosphatase step prior to cloning [29] and while these protocols would clone the small DNA pieces, except for those of repetitive elements, the small DNA pieces would likely be represented at low levels. Regardless, it seems prudent to include a DNase step in future applications.

## Materials and Methods

### Strains and culture

The following strains were used in our studies: *nuc-1(e1392)* X, *nuc-1(n3335)* X, *egl-1(n1084, n3082)* V, *ced-8(n1891)* X, *ced-7(n1892)* III, *ced-5(n1812)* IV, *ced-4(n1162)* III, *ced-3(n717)* IV, *ced-3(n1286)* IV, *ced-3(n2888)* IV, *ced-1(e1735)* I, *cps-6(ok1718)* I, *crn-3(ok2269)* II, *crn-7(ok866)* III, *crn-6(tm0890)* III, *crn-4(tm1415)* X. Strains were grown in liquid culture under standard conditions [30] and embryos isolated from gravid adults by bleaching, as described [31]. For the TdT labeling experiment (Figure 3), gravid adults were isolated by sucrose floatation [32] to remove laid embryos prior to bleaching. Embryos were collected by pelleting at low speed, incubated for 105 min in M9 (20°C), and “total RNA” isolated with Trizol as detailed below.

### Small RNA/DNA analysis

Nucleic acid that contained small DNA pieces was prepared using methods typically used for the isolation of total RNA. “Total RNA” was prepared from embryos or adults by adding 4 volumes of TRIzol (Invitrogen), freezing in liquid nitrogen, followed by isolation according to manufacturer's specifications. In some cases RNA/small DNA was further purified using a mirVana miRNA Isolation Kit (Ambion), with identical results. DNA pieces were visualized with conventional northern analysis protocols. In brief, 40 µg of “total RNA” was electrophoresed on a 17% polyacrylamide (19∶1)/8 M Urea gel at 35W for 1 hr 45 min. Nucleic acid was transferred to Hybond NX membrane (GE-Amersham) using a Biorad wet transfer cell (80V, 1 hr, 0.5X TBE), cross-linked to the membrane using EDC (30 min, 60°C; [33]), and blotted in Ultrahyb-Oligo hybridization buffer (Ambion) at 42°C. Oligonucleotide probes were 5′ end labeled with γ-[^32^P]-(ATP) using T4 polynucleotide kinase. Membranes were washed three times in 2xSSC +0.1% SDS, and exposed on a PhosphorImager screen (Molecular Dynamics).

In some cases nucleic acid was subjected to various treatments prior to electrophoresis. Samples (40 µg) were treated with DNase by incubating with 6U of Turbo DNase (Ambion), followed by organic extraction (Phenol:Chloroform:Isoamyl alcohol, 25∶24∶1), and ethanol precipitation in the presence of GlycoBlue (Ambion). For RNase treatment, nucleic acid (40 µg) was incubated with 10U of RNase A, followed by organic extraction and precipitation as for DNase treated samples. Alkaline hydrolysis was performed by incubating 40 µg of “total RNA” in 20 µl of 0.25 M NaOH (42°C, 16 h). Samples were then diluted to 95 µl with water, neutralized by adding 5 µl of 1 M HCl, and ethanol precipitated in the presence of GlycoBlue.

For labeling with terminal deoxynucleotidyl transferase (TdT), 3 µg “total RNA” was incubated (20 min, 37°C) in 10 µl of 1X terminal transferase buffer (NEB) containing 1 µl α-^32^P Cordycepin 5′ triphosphate (10 µCi, 2 pmoles; Perkin Elmer), 20U TdT (NEB). The reaction was stopped by incubating at 70°C (10 min). Free cordycepin triphosphate was removed by size exclusion using Chromaspin-10 columns (Clontech), and the sample was ethanol precipitated with GlycoBlue, and resuspended in 15 µl water. Reaction products were visualized by electrophoresing 5 µl on a 17% polyacrylamide/8M Urea gel, followed by exposure of the wet gel on a PhosphorImager Screen. CIP treatment was performed by incubating 20 µg “total RNA” in 20 µl of 1X NEB buffer #3 containing 20U CIP (NEB) for 30 min at 37°C, followed by 30 min at 50°C. The samples were then organic extracted, free phosphate was removed by size exclusion using Chromaspin-10 columns, followed by ethanol precipitation in the presence of GlycoBlue, and resuspension in water; 3 µg was then used for the TdT reaction.

### Hybridization probes

#### U6

A mix of 2 oligonucleotides antisense to U6 were used for normalizing blots.

anti-u6 probe1: ctctgtattgttccaattttagtatatgttctcgg

anti-u6 probe2: cacgaatttgcgtgtcatcctt

#### small DNA pieces

Unless specified, small DNA pieces were detected with a mix of the three probes below:

cele42m1: gcacatatctgggtcagatttacggcgcgttgcgtgtcgcgtcgcggctcg

cele14b: TTGTGACGTCAGCACGTTCTTAACCATGCGAAATCAGTTGAGAACTCTGC

*clec-67* UTR2: gcaattgccgaagttgccgattgccgaaaatcaaaattgccgaaaatcta

The same pattern was detected with an antisense probe to *clec-67*:

*clec-67*asUTR2: tagattttcggcaattttgattttcggcaatcggcaacttcggcaattgc

#### Transposons

Probes used to detect Class 1 and Class 2 transposons are specified below with the name of the element, followed by our nomenclature to designate the specific sequence in parentheses.

Class I:

Rte-1(rtp1): TCTGGCCTGCGTGAGACGGTGTACTTCTTGGCCAGATAATTTGCACTTGG

Rte-1(rtp2): GAAAACGTGTTCGAGGCAAGCGGAGAAGAGATTCGGAGAGATGGGATCTCC

Frodo-1(rtp1): TAGCGCAATGAATCTTATTTTGGCACCTCATGTCCTATGCATGTTTAATA

Frodo-1(rtp2): accaaaaccaagttcatctgggggaaatgcaaaaccaaactacgaaggca

Frodo-2(rtp1): cttcttgtttctatccctcttattcagtgtattgtatttatgatctattG

Frodo-2(rtp2): tcctccctcctgacctaactgtcctgttaacaaactttcgttcctattta

Sam1(rtp1): gtagacaataaattaaattgggaaagcctactactgtatgacgagtacgA

Sam1(rtp2): aataattttatggaaggttcggaggatttgaagaacccgtctatggtgg

Sam7(rtp1): atataataatcataggccttcttttagactctgtaattctgataagatcG

Sam7(rtp2): aatggggagtattatgaaaaatagcattccagacggggacagttgaggtg

Sam8(rtp1): tatgtggtgttcaaactttagcttgcaatcagtaattagacctaagtctC

Sam8(rtp2): ggaggagaatgctttgagaatttgtttagacctaagcatggctaggcaac

Sam9(rtp1): gtctggtggagaatagttccatgatgcacagtctgttttcgtaagaggtG

Sam9(rtp2): agtagaaccagtaagaagtctatataatagtttcatttgagctttaattc

Class II:

IR-3(tp1): cattatgaaattcggtgttttcagacaattctgagtctaataaagcaA

IR-3(tp2): aaataccttaataaagctgaaaacgcaagataatcgacatctataatt

Tc4(tp1): cctgcagagatatggagcctcaaaaacaagatgttttgccaatatcgA

Tc4(tp2): ggcgccagagacaaaaatccactgttaaccataggaaatgcactgatttc

Tc6(tp1): tggtgcaataagcggcaggatttgaagcatatcgagtgatcatatccctC

Tc6(tp2): cactgccaaaatcgtattcagtttgctaaaatcagccagagaactaacgg

Tc7(tp1): actttctcaaacctttcattaaaaatatgtttcttgagttacagtcgT

Tc7(tp2): acccaaaacatggcaagtgataaagcggccgacatctcgtgagtccgct

## Supporting Information

Figure S1 Probes for *clec-67* sequences. Nucleotides 1801–2885 of the unspliced *clec-67* mRNA (IV:3922159..3925043) are shown with sequences of the seven probes indicated in capital letters. Probes were designed to detect an antisense sequence and are identical to the mRNA sequence. Probe 5 was designed to hybridize to a spliced sequence and is shown in two pieces separated by intronic sequences. The seventh probe, *clec-67*UTR2, as indicated in Materials and Methods, was routinely used to detect the small DNA pieces. *clec-67*UTR1 and *clec-67*UTR2 overlap a simple repeat (underlined; IV 3924585-4641).
(8.28 MB TIF)

Figure S2 Small DNA pieces are detected with sense or antisense probes to repetitive elements. Various probes as defined in Figure S1 were used for hybridization to blots of “total RNA” prepared from *C. elegans* young adults (A) or embryos (E). Even after overexposure a hybridization signal was not observed with probes to nonrepetitive regions (1–5). In contrast, the small DNA pieces were evident using sense or antisense probes for *clec-67*UTR1 or *clec-67*UTR2. M, RNA decade markers (Ambion).
(6.75 MB TIF)

Figure S3 Characterization of small nucleic acid pieces detected with probes to Class I retrotransposons. “Total RNA” (40 µg) isolated from wildtype *C. elegans* embryos was analyzed by northern analysis using a mixture of 14 radiolabeled oligonucleotides designed to hybridize to Class I retrotransposons (Materials and Methods). Samples were exposed to various treatments (see Figure 1B) prior to northern analysis. D, DNase treatment; R, RNase treatment, AH, alkaline hydrolysis. M, 10 bp DNA ladder (Invitrogen). All bands were sensitive to DNase treatment, except an ∼28 nt band (asterisk). Nucleic acid species detected with retrotransposon probes are low abundance, necessitating long exposure times that lead to poor resolution. In subsequent experiments (e.g., Figure 1C), 100 µg of “total RNA” was subjected to the mirVana protocol to enrich for small nucleic acid species, resulting in northern analyses of higher resolution.
(2.84 MB TIF)

Figure S4 Additional alleles of *ced-3* mutant strains lack small DNA pieces. “Total RNA” isolated from embryos of *ced-3* or wildtype (N2) *C. elegans* was analyzed by northern blot using a mixture of three different probes as detailed (see Materials and Methods). M, 10 bp DNA ladder (Invitrogen).
(4.64 MB TIF)
