# Supplementary figures and images for: Small DNA Pieces in C. elegans Are Intermediates of DNA Fragmentation during Apoptosis
Source: PLoS One. 2010 Jun 18;5(6):e11217. doi: 10.1371/journal.pone.0011217 (PMC2887891; doi:10.1371/journal.pone.0011217)

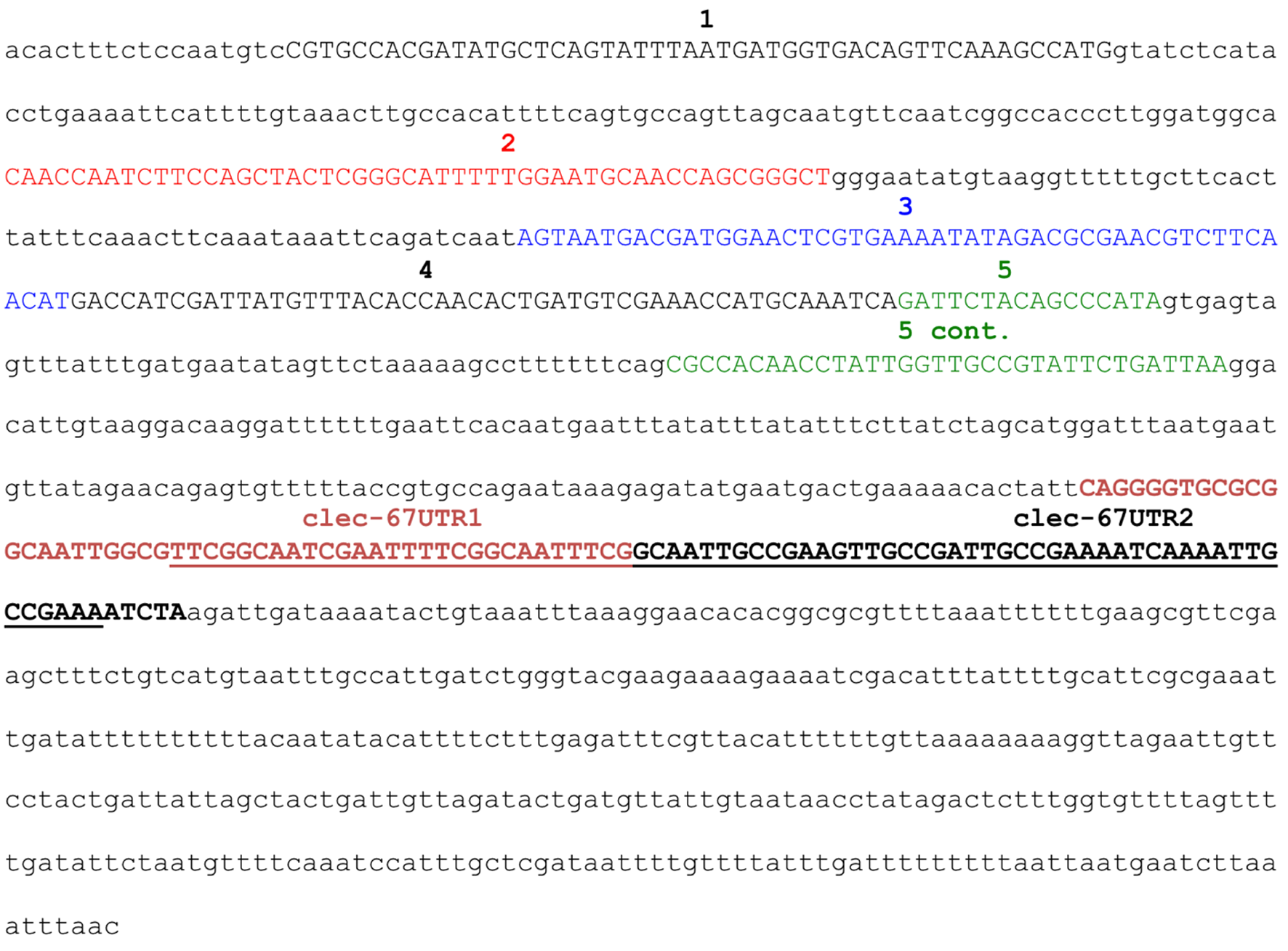

Supplement: Figure S1 — Probes for clec-67 sequences. Nucleotides 1801–2885 of the unspliced clec-67 mRNA (IV:3922159..3925043) are shown with sequences of the seven probes indicated in capital letters. Probes were designed to detect an antisense sequence and are identical to the mRNA sequence. Probe 5 was designed to hybridize to a spliced sequence and is shown in two pieces separated by intronic sequences. The seventh probe, clec-67UTR2, as indicated in Materials and Methods, was routinely used to detect the small DNA pieces. clec-67UTR1 and clec-67UTR2 overlap a simple repeat (underlined; IV 3924585-4641). (8.28 MB TIF) [file pone.0011217.s001.tif]

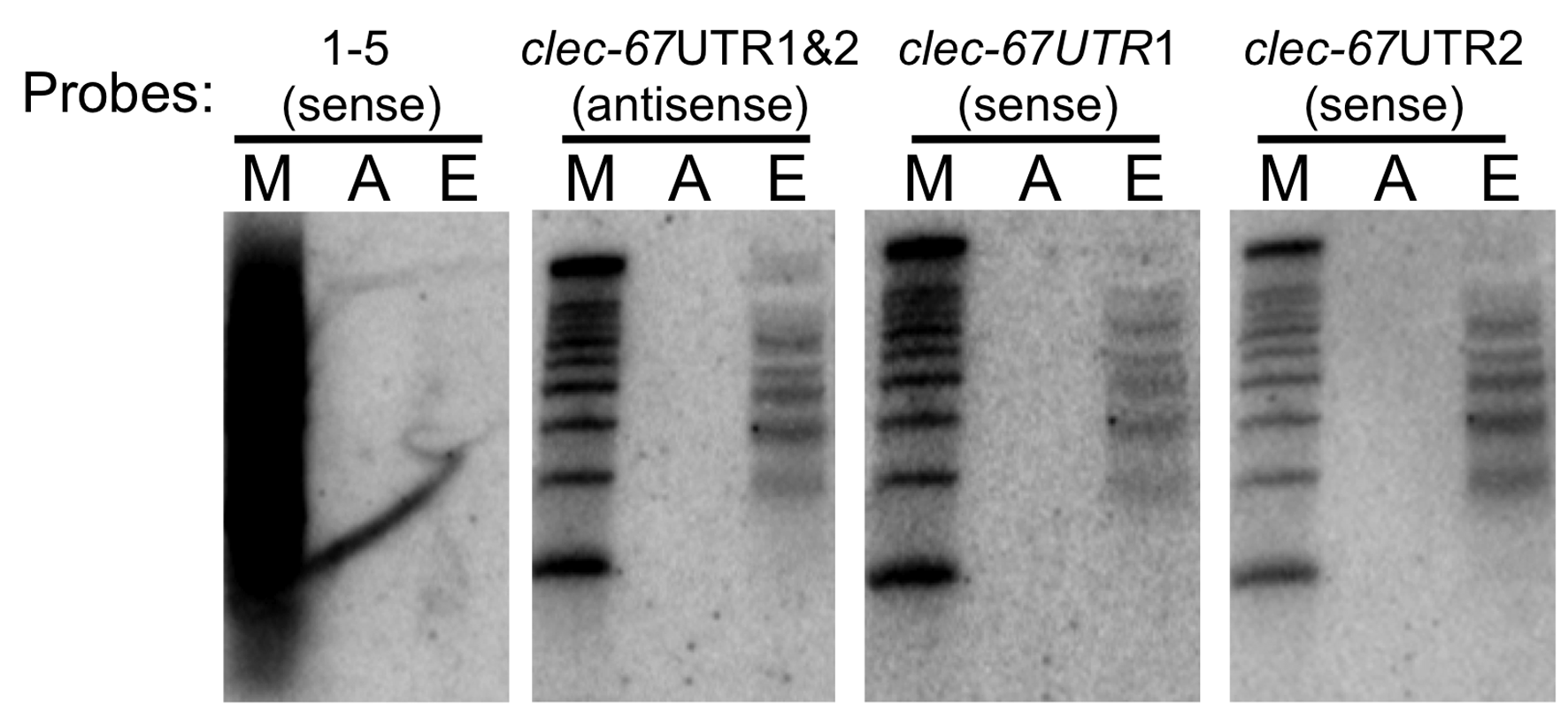

Supplement: Figure S2 — Small DNA pieces are detected with sense or antisense probes to repetitive elements. Various probes as defined in Figure S1 were used for hybridization to blots of “total RNA” prepared from C. elegans young adults (A) or embryos (E). Even after overexposure a hybridization signal was not observed with probes to nonrepetitive regions (1–5). In contrast, the small DNA pieces were evident using sense or antisense probes for clec-67UTR1 or clec-67UTR2. M, RNA decade markers (Ambion). (6.75 MB TIF) [file pone.0011217.s002.tif]

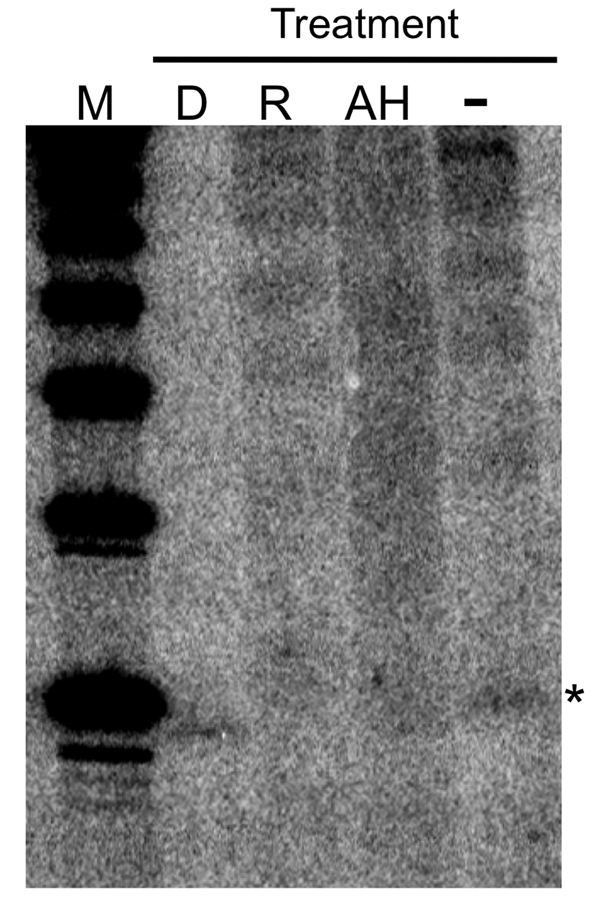

Supplement: Figure S3 — Characterization of small nucleic acid pieces detected with probes to Class I retrotransposons. “Total RNA” (40 µg) isolated from wildtype C. elegans embryos was analyzed by northern analysis using a mixture of 14 radiolabeled oligonucleotides designed to hybridize to Class I retrotransposons (Materials and Methods). Samples were exposed to various treatments (see Figure 1B) prior to northern analysis. D, DNase treatment; R, RNase treatment, AH, alkaline hydrolysis. M, 10 bp DNA ladder (Invitrogen). All bands were sensitive to DNase treatment, except an ∼28 nt band (asterisk). Nucleic acid species detected with retrotransposon probes are low abundance, necessitating long exposure times that lead to poor resolution. In subsequent experiments (e.g., Figure 1C), 100 µg of “total RNA” was subjected to the mirVana protocol to enrich for small nucleic acid species, resulting in northern analyses of higher resolution. (2.84 MB TIF) [file pone.0011217.s003.tif]

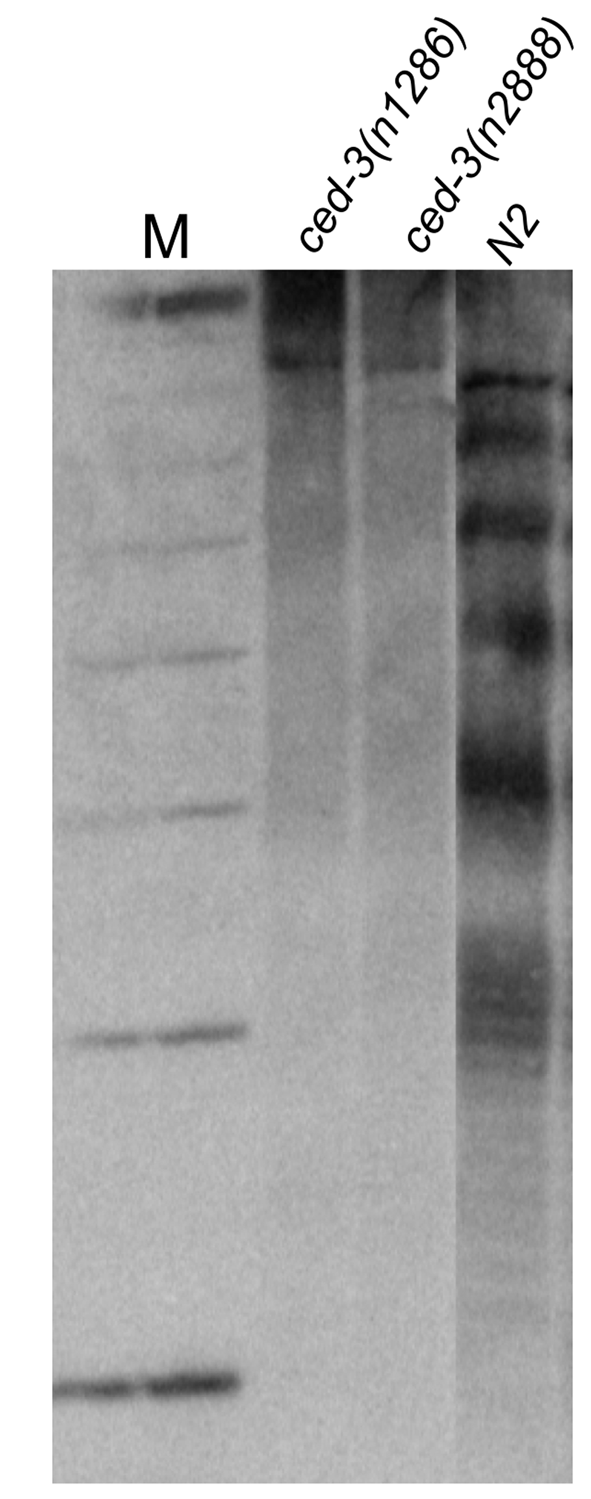

Supplement: Figure S4 — Additional alleles of ced-3 mutant strains lack small DNA pieces. “Total RNA” isolated from embryos of ced-3 or wildtype (N2) C. elegans was analyzed by northern blot using a mixture of three different probes as detailed (see Materials and Methods). M, 10 bp DNA ladder (Invitrogen). (4.64 MB TIF) [file pone.0011217.s004.tif]
